# Fertilization and Cytogenetic Examination of Interspecific Reciprocal Hybridization between the Scallops, *Chlamys farreri* and *Mimachlamys nobilis*

**DOI:** 10.1371/journal.pone.0027235

**Published:** 2011-11-14

**Authors:** Xiaoting Huang, Ke Bi, Liping Hu, Yan Sun, Wei Lu, Zhenmin Bao

**Affiliations:** 1 Key Laboratory of Marine Genetics and Breeding (MGB), Ministry of Education, College of Marine Life Sciences, Ocean University of China, Qingdao, China; 2 Museum of Vertebrate Zoology, University of California, Berkeley, California, United States of America; Auburn University, United States of America

## Abstract

Crossbreeding is a powerful tool for improving productivity and profitability in aquaculture. We conducted a pilot study of an artificial cross between two important cultivated scallops in China, *Chlamys farreri* and *Mimachlamys nobilis*, to test the feasibility of interspecific hybridization. Reciprocal hybridization experiments were performed using a single-pair mating strategy (*M. nobilis* ♀ × *C. farreri* ♂ and *C. farreri* ♀ × *M. nobilis* ♂). The fertilization of each pair was tracked using fluorescence staining of the gametes, and the chromosomes of the F1 hybrid larvae were examined via conventional karyotyping and genomic *in situ* hybridization (GISH). We observed moderate fertilization success in both interspecific crosses, although the overall fertilization was generally less rapid than that of intraspecific crosses. Conventional karyotyping showed that 70.4% of the viable F1 larvae in *M. nobilis* ♀ × *C. farreri* ♂ and 55.4% in *C. farreri* ♀ × *M. nobilis* ♂ comprised hybrid karyotypes (2n = 35 = 6m+5sm+11st+13t), and the results were further confirmed by GISH. Interestingly, we detected a few F1 from the *M. nobilis* ♀ × *C. farreri* ♂ cross that appeared to have developed gynogenetically. In addition, chromosome fragmentations, aneuploids and allopolyploids were observed in some F1 individuals. Our study presents evidence that the artificial cross between *M. nobilis* and *C. farreri* is experimentally possible. Further investigations of the potential heterosis of the viable F1 offspring at various developmental stages should be conducted to obtain a comprehensive evaluation of the feasibility of crossbreeding between these two scallop species.

## Introduction

Heterosis (hybrid vigor), defined as an increase in the performance (e.g., the growth rate, output, and tolerance of environmental extremes) of hybrids over both parental species, has been widely tested or established in many commercial species in aquaculture [1]–[6]. Crossbreeding has been shown to be effective for improving the productivity and disease resistance of some scallops. For example, Bower et al. [7] reported that hybrid scallops from *Patinopecten yessoensis* × *P. caurinus* possessed high heterosis that shows a resistance to infection by *Perkinsus qugwadi*, a lethal pathogen of *P. yessoensis*. Cruz and Ibarra [8] compared the growth and survival of two stocks of the catarina scallop (*Argopecten circularis*), as well as for their reciprocal crosses, and described a strong maternal effects and heterosis in the hybrids. Yang et al. [9] found that the hybrid offspring of *Chlamys farreri* and *P. yessoensis* possessed heterosis in both growth and disease resistance. Wang et al. [10] performed reciprocal hybridization between Peruvian scallop, *A. purpuratus*, and bay scallop, *A. irradians*. The former one distributes naturally along the Pacific coasts of South America and was introduced to China 2007 and 2008, while the latter one distributes naturally along the Atlantic coast from New Jersey up north to Cape Cod in eastern North America and was introduced to China in 1982. Both of them showed similar karyotypes (2n = 32 = 5st + 11t). The hybrids of these two *Argopecten* scallops exhibited great increase in production traits as well as some other interesting new characteristics.

*C. farreri* and *Mimachlamys nobilis* are two of the most important cultivated marine scallop species in China. With the increased density of mariculture in recent years, the majority of stocks have demonstrated slow growth and/or experienced extensive mortalities, thereby, posing a serious threat to the Chinese scallop industry [11], [12]. These two scallops are distinct in geographic distributions and thermal preference. *C. farreri* is commonly found along the sea coasts of northern China, Japan, Korea and Sakhalin in Russia, while *M. nobilis* distributes along the coasts of the southern China and Japan. *C. farreri* lives in temperatures ranging from 15 to 25°C, but *M. noblilis* survives with warmer temperature range, from 18 to 30°C [13]. These two species also have highly divergent genomes [14], [15] and karyotypes [16], [17]. Because of this ecological and genetic heterogeneity, *C. farreri* and *M. nobilis* may potentially represent a good model system for testing the feasibility of crossbreeding to obtain desirable scallop breeds (i.e., desirable hybrids with wider thermal tolerances). Recently, single-pair reciprocal crosses between *C. farreri* and *M. nobilis* were artificially performed. To verify the identity of the F1 hybrids, we used genomic *in situ* hybridization (GISH) to examine the genomic constitution of the larvae. GISH is one of the most useful techniques for hybrid identification and was first established for plants by Schwarzacher et al. [18]. This technique uses the total genomic DNA from one species as the probes labeled with fluorescent dyes in *in situ* hybridization [19], [20]. Excess amounts of unlabeled total genomic DNA from another species are simultaneously used as blocking DNA to increase the specificity of probes and to ensure the discrimination of genomes/chromosomes from related species. Under a fluorescence microscope, the chromosomes with fluorescent - labeled probes differentially compared to the ones with unrelated DNA sequences. These particular features of GISH make it a powerful tool for analyzing interspecific and intergeneric hybrids and allopolyploid species as well as introgression, addition and substitution lines [20], [21].

The objectives of the present study were to examine the fertilization in the reciprocal hybridization of *C. farreri* and *M. nobilis* and to identity the F1 hybrids from each artificial cross. A variety of experimental approaches, including gametic fluorescence staining, conventional karyotyping and GISH, were applied to examine the cytological process of fertilization and the genomic compositions of the F1 hybrids and to evaluate the feasibility of artificial hybridization between these two scallop species.

## Materials and Methods

### Artificial hybridization

Sexually mature *C. farreri* and *M. nobilis* (approximately two years old) were collected from the Xunshan scallop hatchery in Shandong Province, China. *C. farreri* and *M. nobilis* were induced to ovulate and the insemination was performed according to the method outlined by Wang and Wang [13]. Artificial crosses were conducted with the following four different combinations: *M. nobilis* ♀ × *C. farreri* ♂, *C. farreri* ♀ × *M. nobilis* ♂, *M. nobilis* ♀ × *M. nobilis* ♂, and *C. farreri* ♀ × *C. farreri* ♂. All of the crosses were performed based on a single-pair mating scheme (one male to one female).

### Observation of fertilization by fluorescent staining

The fertilized eggs and developing embryos from each cross were sampled every 5 minutes from the initial time of the mixture of sperm and eggs, fixed with 2% glutaraldehyde and 2.5% paraformaldehyde in phosphate-buffer saline (10 µM PBS, pH = 7.4) and stored at 4°C until use. Prior to the examination, the eggs or embryos were stained with Hoechst 33258 (Molecular Probes) for 5 to 10 minutes in the dark and observed with a Nikon E-600 fluorescent microscope equipped with an appropriate filter for Hoechst 33258. Digital images were recorded using a CCD camera (COHU).

### Chromosome preparation and karyotyping

Chromosome preparations from the adult scallops were made using the protocol established by Wang et al. [17]. To obtain the chromosomes of the hybrids, the trochophores were collected and cultured in 0.01% colchicine at room temperature for 2 hours. The larvae were then exposed to 0.075 M KCl solution for 30 minutes, fixed with Carnoy's fixative (ethanol:glacial acetic acid = 3∶1), and stored at −20°C until use. The fixed larvae were dissociated into fine pieces by gentle pipetting in 50% acetic acid solution and the resulting cell suspension was dropped onto a pre-heated glass slide and air-dried. The chromosome preparations for GISH were preserved at −20°C until use. The preparations for the traditional karyotyping were stained with 5% Giemsa's solution in phosphate-buffer (pH = 7.4) for 20 minutes and photographed using a Nikon E-600 microscope. The karyotype was determined from more than 10 good metaphase plates and classified according to the criteria defined by Levan et al. [22].

### Genomic *in situ* hybridization (GISH)

Total genomic DNA from *C. farreri* and *M. nobilis* was isolated from the adductor muscle using a standard phenol/chloroform extraction protocol [23]. The genomic DNA probes were labeled with biotin-11-dUTP by nick translation kit (Roche) following the manufacturer's protocol. The biotinylated probes were resolved at a concentration of 5 ng/µl in a hybridization solution of 2× SSC, 50% deionized formamide (Shanghai Sangon), 10% dextran sulfate salt (Shanghai Sangon), 1× Denhardt's (Shanghai Sangon), 0.1% SDS (BBI) and 500 ng/µl salmon testis DNA (Amresco). Unlabeled blocking DNA was made by autoclaving DNA into fragments of approximately 100–200 bp in length. The blocking DNA was added to the probe solution. The ratio of genomic probes/blocking DNA (P/B) was optimized using a range from 1∶10 to 1∶30. The identification of the chromosomal composition in the hybrids was analyzed using GISH, with the protocol described by Bi and Bogart [24]. The hybridization signals were detected using a Nikon E-600 fluorescent microscope equipped with the appropriate filters for FITC and PI. The digital images were recorded using a CCD camera and analyzed with a Lucia-FISH Image System under the default settings. At least 50 metaphases were examined for each sample.

## Results and Discussion

### Fertilization of interspecific hybridization

**Figure 1:**
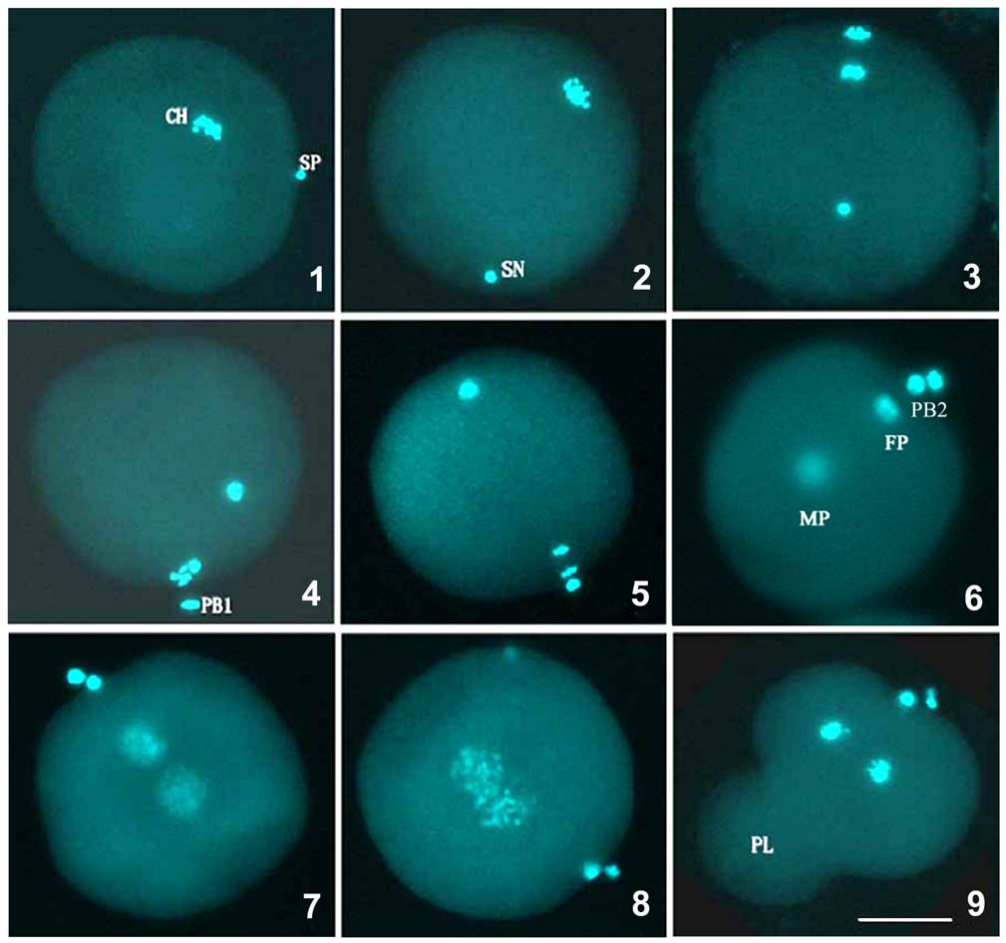
Cytological observation of the fertilization in *M. nobilis* ♀ × C. farreri ♂ crosses by fluorescent microscope. (1) Sperm bound to the egg. (2) Sperm penetrating the egg. (3) Meiotic anaphase I of the egg. (4) Release of the first polar body. (5) Meiotic anaphase II of the egg. (6) Release of the second polar body; female and male pronuclei are formed. (7, 8) Fusion of female and male pronuclei. (9) Formation of polar lobe and the first mitotic division. SP: Sperm. CH: Chromosome. SN: Sperm nucleus. PB1: First polar body. PB2: Second polar body. MP: Male pronucleus. FP: Female pronucleus. PL: Polar lobe. Bar = 20 µm.

The fertilization of the four artificial crosses was examined by the fluorescence staining of the nuclei of the gametes and early embryos. As an example, the detailed fertilization process of *M. nobilis* ♀ × *C. farreri* ♂ is shown in Fig. 1. The mature eggs of *M. nobilis* and *C. farreri* were 60–70 µm in diameter and remained at the metaphase stage of meiosis I. The sperm nuclei produced by both species were approximately 1 µm in diameter. In both of the interspecific crosses (*M. nobilis* ♀ × *C. farreri* ♂ and *C. farreri* ♀ × *M. nobilis*♂), the sperm did not bind to and penetrate the eggs as rapidly as they did in the two intraspecific crosses (*M. nobilis* ♀ × *M. nobilis* ♂ and *C. farreri* ♀ × *C. farreri* ♂). Under the activation of the dispersed sperm nucleus, the eggs released their first polar body (PB1) and second polar body (PB2) successively and completed the meiotic divisions. The male and female pronuclei were then formed when the PB2 was released. The two pronuclei expanded and moved towards each other, fused, and initiated the mitosis of the first cleavage. In both interspecific crosses, a small portion of the fertilized eggs was found to be misshapen, and most of these misshapen eggs showed a high mortality rate during embryogenesis. Polyspermy was not detected during fertilization.

It is probable that the divergent genomes and chromosomes of *C. farreri* and *M. nobilis* cause hybrid lethality and sterility, thereby, producing a post-zygotic reproductive barrier between the two species. Furthermore, in many marine invertebrates, fertilization is accomplished externally. These organisms may have evolved cellular and molecular mechanisms to ensure species-specificity in gamete recognition and interactions [25]. However, previous results showed that such reproductive barriers were not evident in at least 51% of the hybrids in *C. farreri* ♀ × *M. nobilis* ♂ and 82% in *M. nobilis* ♀ × *C. farreri* ♂ [26], [27]. The observations of the development of the early embryos and juveniles suggest that the majority of the F1 hybrids were viable, although it is probable that they would be sterile at maturity owing to their unbalanced chromosome numbers. In fact, we found that the time required for the recognition and reaction between the heterospecific gametes was much longer than that found for the intraspecific crosses. This result suggested that interspecific gametic incompatibility is indeed present but is imperfect. The extent of the incompatibility also differed between the two interspecific crosses, as shown by their different fertilization rates (51% vs. 82%). A differential fertilization rate between reciprocal crosses has also been observed in the artificial hybridization of some fish species [28] and the scallops, *C. farreri* and *P. yessoensis* [29]. A plausible explanation of these observations might be that there is less compatibility between the *M. nobilis* nuclear genome and the *C. farreri* cytoplasm than between the *C. farreri* nuclear genome and the *M. nobilis* cytoplasm.

### Karyotypic and GISH identification of interspecific hybrids

**Figure 2:**
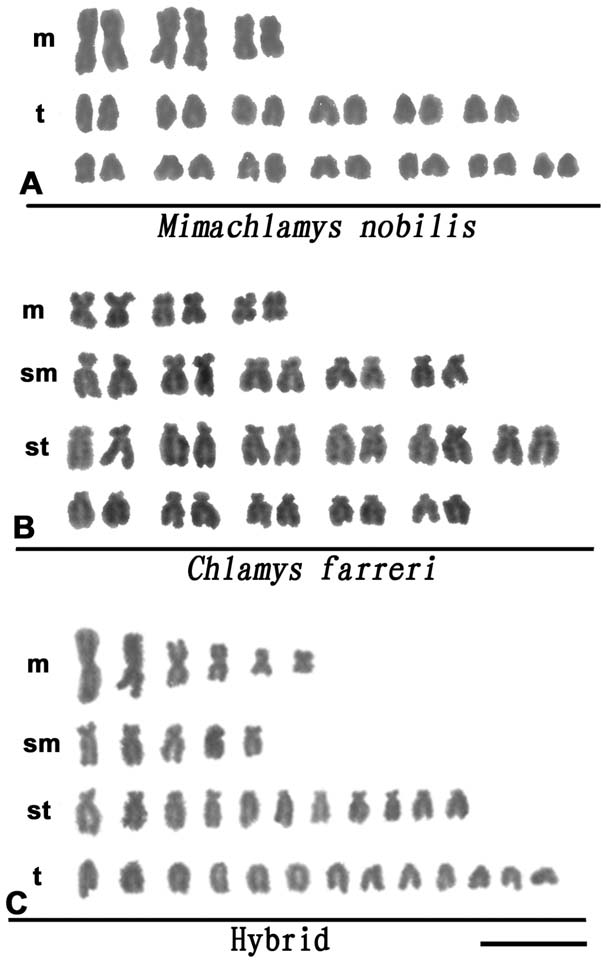
Chromosomes and karyotypes of *M. nobilis* (A), *C. farreri* (B), and the F1 hybrids (C). **Bar  =** **5 µm.**

The karyotype of the offspring from each cross was classified using the method described by Levan et al. [22]. The karyotype of *M. nobilis* was 2n = 32 = 6m+26t (Fig. 2A), and the karyotype of *C. farreri* was 2n = 38 = 6m+10sm+22st (Fig. 2B). These results are consistent with those previously reported [16], [17]. The chromosomes of both species were generally small with an average of less than 2 µm in length, and no satellites or secondary constrictions were detected. The karyotype of *M. nobilis* was characterized by its three long, metacentric chromosomes, with relative lengths of 13.03, 11.52 and 7.53, respectively, whereas the relative lengths of the remaining telocentric chromosomes ranged from 3.66 to 6.67 (Table S1). Compared with *M. nobilis*, *C. farreri* didn't have telocentric chromosomes, its chromosomes were relatively small, and the relative lengths of the 38 chromosomes did not vary drastically. Most of the hybrids in the two interspecific crosses (70.4% in *M. nobilis* ♀ × *C. farrer* ♂ and 55.4% in *C. farreri* ♀ × *M. nobilis* ♂) exhibited 35 chromosomes per cell (Table 1). The karyotype of the hybrids from both crosses was composed of 6m+5sm+11st+13t (Fig. 2C). The number and morphology of the chromosomes in these hybrids indicated that they received one haploid genome from each parent.

**Table 1:** Chromosome counts of scallop hybrids and their parents.

*M. nobilis* ♀	Chromosome number	≤30	31	32	33	34	35	36	37	≥38	total
×	Cell number	5	4	101	3	1	3	2	3	0	122
*M. nobilis* ♂	Frequency (%)	4.1	3.3	82.7	2.5	0.8	2.5	1.6	2.5	0	100
*C. farreri* ♀	Chromosome number	≤32	33	34	35	36	37	38	39	≥40	total
×	Cell number	2	3	1	2	0	4	97	6	0	115
*C. farreri* ♂	Frequency (%)	1.7	2.6	0.9	1.7	0	3.5	84.4	5.2	0	100
*M. nobilis* ♀	Chromosome number	≤30	31	32	33	34	35	36	37	≥38	total
×	Cell number	4	3	21	4	7	117	2	3	5	166
*C. farreri* ♂	Frequency (%)	2.4	1.8	12.7	2.4	4.2	70.4	1.3	1.8	3.0	100
*C. farreri* ♀	Chromosome number	≤30	31	32	33	34	35	36	37	≥38	total
×	Cell number	7	6	5	9	4	62	2	5	12	112
*M. nobilis* ♂	Frequency (%)	6.2	5.3	4.5	8.0	3.6	55.4	1.8	4.5	10.7	100

**Figure 3:**
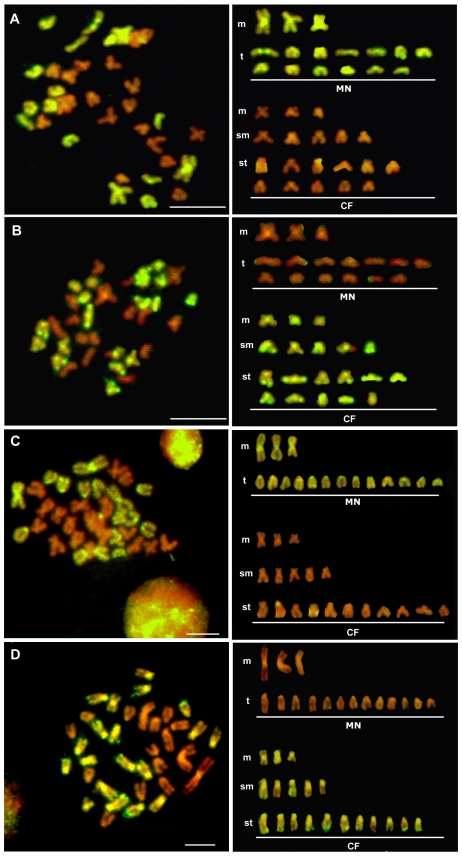
Representative metaphase chromosomes and karyotypes of the F1 hybrids of *M. nobilis* ♀ × *C. farreri* ♂ (A, B) and *C. farreri* ♀× *M. nobilis* ♂ (C, D) examined by GISH. CF: chromosomes from *C. farreri*. MN: chromosomes from *M. nobilis*. Chromosomes are painted by FITC (green) and counterstained by PI (red). In (A, C), the chromosomes originated from *M. nobilis* are identified in green using the labeled genome DNA probes from *M. nobilis*. In (B, D), the chromosomes from *C. farreri* are identified in green using the labeled genome DNA probes from *C. farreri*. Bars = 5 µm.

With a P/B ratio of 1∶10, the GISH technique effectively distinguished all of the chromosomes inherited from *M. nobilis* and *C. farreri* in the hybrid genomes. The chromosomes from *M. nobilis* and *C. farreri* were identified with distinct fluorescence signals and confirmed the results obtained by the conventional karyotyping (Fig. 3). The majority of the hybrids in both crosses contained 35 chromosomes, the sum of the chromosomes number of the haploid genomes of *M. nobilis* (n = 16) and *C. farreri* (n = 19). The fluorescence signals were not uniform across the chromosomes, as noted in nearly all of the metaphases that we examined. This phenomenon was found among the different chromosomes and also along the same individual chromosomes (Fig. 3). An increased concentration of blocking DNA failed to promote a uniform hybridization of the probes over the complete length of the chromosomes. Because GISH is a method based primarily on the differences in repetitive sequences, this phenomenon might be initiated by the uneven clustering of repetitive sequences along the chromosome arms or on different chromosomes in the same cell. In our experiments, the hybridization signals were found to be stronger in the chromosomal peri-centromeric and/or centromeric heterochromatin regions than in the chromosome arms. This finding could reflect a more rapid rate of evolution and divergence in the heterochromatin regions [30], where species-specific repetitive DNA accumulates rapidly in closely related species [31], [32]. As a result, the blocking DNA did not effectively block these regions. Strong GISH signals were also discovered in the telomeric and/or peri-telomeric regions of some of the chromosomes, especially in *C. farreri* (Fig. 3B and 3D). Similarly, we hypothesized that the highly conserved heterochromatin sequences clustered in these regions may have produced this phenomenon. Direct evidence for this hypothesis was reported in a sequencing study of a fosmid library of *C. farreri* [33] in which various species-specific DNA satellites were found in the genome. These specific sequences were mapped to the peri-telomeric regions of 12–13 pairs of chromosomes of *C. farreri* via fluorescence *in situ* hybridization (FISH) [34], a result concordant with the strong signals that we observed in the GISH experiments.

### Rare gynogen-like offspring initiated by the interspecific cross of *M. nobilis* ♀ × *C. farreri* ♂

**Figure 4:**
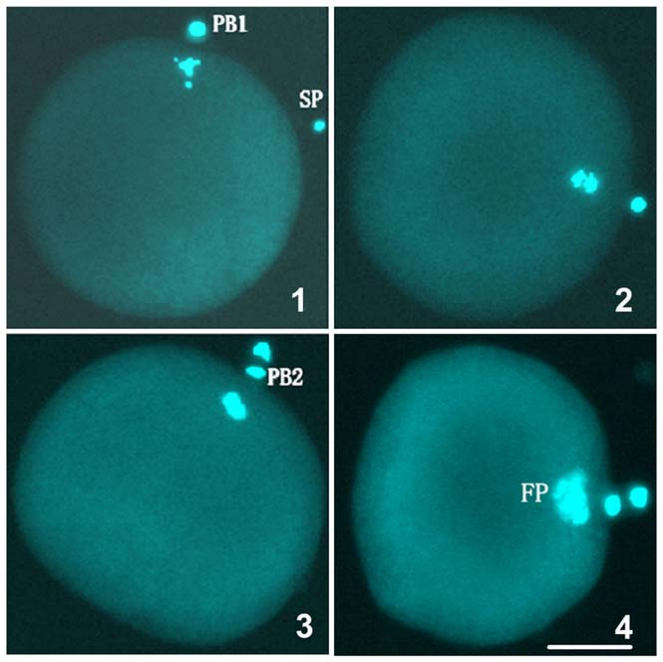
Observations of gynogenetic eggs derived from the interspecific cross of *M. nobilis* ♀ × *C. farreri* ♂. (1) The egg has released the first polar body with a sperm binding to it. (2) The egg proceeds to meiotic anaphase II without incorporating a sperm nucleus. (3, 4) The egg releases the second polar body without incorporating a sperm nucleus. SP: Sperm. PB1: First polar body. PB2: Second polar body. FP: Female pronucleus. Bar = 20 µm.

**Figure 5:**
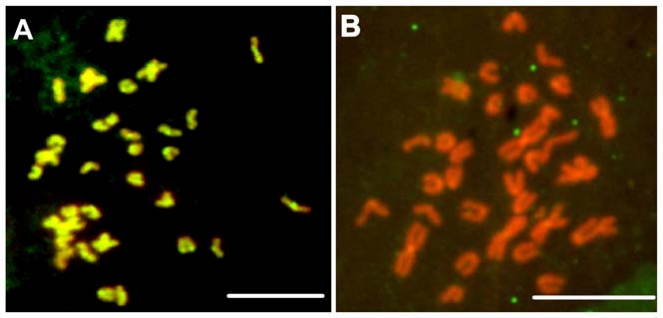
Chromosomes of a gynogen-like hybrid examined by GISH. (A) All of the chromosomes are painted green using the *M. nobilis* genomic DNA probes. (B) No *C. farreri* chromosomes or chromosome segments were found when using the *C. farreri* genomic DNA as probes. Bars = 5 µm.

During the examination of the fertilization of *M. nobilis ♀* × *C. farreri ♂*, we found that a few of the *M. nobilis* eggs appeared to proceed to the first cleavage without the incorporation of sperm (Fig. 4). A similar phenomenon was not detected in either the intraspecific crosses or in the other interspecific cross, *C. farreri ♀* × *M. nobilis ♂*. Although the majority of the F1 hybrids contained 35 chromosomes, approximately 12.7% of the larvae from the *M. nobilis ♀* × *C. farreri ♂* cross that we examined showed a karyotype identical to that of *M. nobilis*, 2n = 32 = 6m+26t. No haploids were found. This discovery was further verified by the GISH analysis. In these metaphase spreads, all of the chromosomes were painted with the *M. nobilis* genomic DNA probes (Fig. 5A). For the artificial crosses, we used a single-pair mating strategy, and this approach was essential to ensure complete interspecific hybridization and to prevent contamination by the gametes from unexpected individuals/species. The maternal *M. nobilis* was carefully examined and shown not to be a hermaphrodite. This result suggested that these gynogen-like offspring might be generated by a cryptic mechanism during interspecific hybridization. We hypothesize that the *C. farreri* genome does not contribute to these gynogen-like progeny and that the sperm from the parental *C. farreri* may have only functioned as stimulators to initiate the further development of *M. nobilis* eggs (gynogenesis). After activation, the eggs resumed their first and second meiotic divisions, released two polar bodies, and proceeded with the process of embryogenesis. The gynogen-like offspring were also observed in hybrids between *C. farreri ♀* × *P. yessoensis ♂*, and this observation was explained by the asynchronous behaviors during mitosis and the replacement of the paternal chromosomes [35].

The artificial induction of gynogenesis in mollusks is of interest to both commercial aquaculturists and researchers in the study of developmental biology and genetics [6]. With the exception of the results obtained by traditional methods, such as cold shock accompanied by the stimulation with UV-inactivated sperm [36], [37], gynogenesis initiated by interspecific hybridization has only been reported in unisexual vertebrates [38]. Unisexual females may undergo a chromosome self-duplication event during the last mitosis prior to the meiotic division during oogenesis, a process termed “premeiotic endomitosis” [38]–[40]. The eggs are unreduced after meiotic division and are genetically identical to their maternal parent. The sperm produced by sympatric sexual males can, subsequently, activate the further development of the unreduced eggs [41]. Because no haploids were found in the hybrid larvae, we speculated that a cryptic chromosome-doubling event might have occurred prior to the first cleavage. An alternative hypothesis is that fusion of the female pronucleus with the PB2 rather than with the sperm nucleus took place in some eggs. It was surprising that we did not find any triploid in the offspring. Both mechanisms may be necessary only for the maintenance of diploidy but could not satisfactorily explain the rejection of sperm nucleus to increase the ploidy level (2n to 3n), and the initiation of a gynogenetic mode of development. Furthermore, if these gynogen-like offspring were derived entirely from their maternal parent, we would expect that all of the loci in their nuclear genomes to be homozygous. Further molecular studies on the F1 hybrids, such as DNA microsatellite diagnoses, can be used to test this prediction.

### Chromosome elimination and rare allopolyploids in the interspecific hybrids

**Figure 6:**
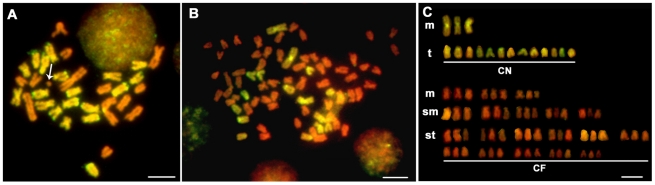
Examples of chromosome elimination (A) and an allotetraploid (B, C) in the F1 hybrids. (A) Chromosomes are identified by GISH using the *C. farreri* genomic DNA probes. The green chromosomes are from *C. farreri*, and the red chromosomes are from *M. nobilis*. Some chromosomes from *M. nobilis* were eliminated in the metaphase spread. A chromosome fragment originating from *M. nobilis* is marked with a single arrow. (B, C) Chromosomes are identified by GISH using the *M. nobilis* genomic DNA probes. The 16 (n) chromosomes originating from *M. nobilis* are shown with FITC fluorescence (green), and the 57 (3n) chromosomes originating from *C. farreri* are counterstained with PI (red). Bars = 5 µm.

In contrast with the interspecific cross of *M. nobilis* ♀ × *C. farreri* ♂, we observed no gynogen-like hybrids of *C. farreri* ♀ × *M. nobilis* ♂. However, we found many cases of aneuploidy, with chromosome number either greater or fewer than the expected diploid chromosome numbers of the hybrid genome (2n = 35). About 44.6% of cells from *C. farreri* ♀ × *M. nobilis* ♂ and 29.6% from *M. nobilis* ♀ × *C. farreri* ♂ were aneuploids, much higher than the intraspecific cross groups: 17.3% for *M. nobilis* and 15.6% for *C. farreri*, indicating the instability of the hybrid genome. However, we cannot rule out that some of the aneuploids could also result from technical shortcomings in chromosome preparation as described in other shellfish [42], [43]. As shown in Fig. 6A, chromosome elimination and fragmentation in some of the hybrids were found. In the current study, whole chromosome eliminations were more commonly observed than the fragmentations and the majority of the eliminated chromosomes were derived from the *M. nobilis* genome rather than the *C. farreri* genome according to GISH results. In addition, we found that more than 16% of hybrid individuals contained metaphases with chromosome numbers greater than 35 (Table 1) and up to 75 in some cases. For example, using GISH, we found that 1–2% of the metaphases consisted of one *M. nobilis* chromosome set and three *C. farreri* chromosome sets (Fig. 6B and 6C). These hybrids appear to include both diploid and polyploid cells to form a mosaic genome.

Chromosome abnormality is known to be one of the causes of hybrid lethality. It is probable that such lethality is induced by a genetic incompatibility between the paternal genome and maternal cytoplasm [44]. Chromosome elimination has been observed in the natural hybrids of some insects, such as the genus, *Nasonia*. This outcome might be influenced by the ratio of parental nuclear genomes and/or cytoplasm and might always cause chromosomes from one parent to be eliminated by their asynchronous behaviors during mitosis [45], [46]. It is possible that the chromosome elimination and fragmentations observed in the present study were associated with a similar mechanism because the cell cycle of *M. nobilis* is generally shorter than that of *C. farreri* [13]. As a result, the paternal genomic components derived from *M. nobilis* were more frequently eliminated during mitosis (Fig. 6A).

Polyploidy, frequently termed “whole genome duplication”, is a major force in the evolution of many eukaryotes, and most angiosperm species have undergone at least one round of polyploidy in their evolutionary history [47]. Allopolyploidy is prevalent in plants and is usually associated with an obvious hybrid vigor. It is rare in animals, and is often associated with unisexuality in vertebrates [38]. In this study, the exact timing and mechanism of the alloploidization events that we observed are unclear. It is possible that the allotetraploids are produced by accidental inhibition of PB1 and subsequent release of one set of chromosomes as “PB2”, or by fusion of the female pronucleus with PB1 and the male pronucleus. Similar phenomenon has been documented in *C. farreri* that PB1 inhibition results in complicated segregation patterns that lead to the formation of triploids, tetraploids, pentaploids, and a wide range of aneuploids [48]. Owing to the rare occurrence, cryptic mechanisms, and unknown fate of the self-chromosome duplications, it is difficult to predict whether and how such events would influence the performance of hybrid scallops.

In conclusion, our study demonstrates that interspecific hybridization between two scallops is experimentally possible. The majority of the hybrid larvae from the two crosses were viable and contained the expected chromosome constituents. Gynogens, allopolyploids and aneuploids were detected in a small portion of the offspring, but their rare occurrence implies that they are unlikely to play a major role in contributing to the overall heterosis of the hybrids. Although these two species can interbreed reciprocally, whether their offspring will achieve heterosis or will actually be subject to hybrid depression requires further investigation. For example, future studies should compare the growth and survival rate, shell size, body weight, resistance to pathogens and temperature fluctuations, and other desired characteristics between the hybrids and their parents.

## Supporting Information

Table S1 Karyotype analysis of 10 metaphases in *M. nobilis*.
(DOC)
