# Supplementary material for: Fertilization and Cytogenetic Examination of Interspecific Reciprocal Hybridization between the Scallops, Chlamys farreri and Mimachlamys nobilis
Source: PLoS One. 2011 Nov 14;6(11):e27235. doi: 10.1371/journal.pone.0027235 (PMC3215693; doi:10.1371/journal.pone.0027235)
Supplement: Table S1 — Karyotype analysis of 10 metaphases in M. nobilis. (DOC) [file pone.0027235.s001.doc]

Table S1. Karyotype analysis of 10 metaphases in *M. nobilis.*

| Group | Chromosome pair No. | Relative length ($\mathop{\bar{X}}\limits\pm S.D$) | Arm ratio ($\mathop{\bar{X}}\limits\pm S.D$) | Type* |
| --- | --- | --- | --- | --- |
| Ⅰ | 1 | 13.03±0.53 | 1.16±0.14 | m |
|  | 2 | 11.52±0.54 | 1.02±0.04 | m |
|  | 3 | 7.53±0.25 | 1.08±0.05 | m |
| Ⅱ | 4 | 6.67±0.24 | ∞ | t |
|  | 5 | 6.08±0.20 | ∞ | t |
|  | 6 | 5.97±0.12 | ∞ | t |
|  | 7 | 5.87±0.10 | ∞ | t |
|  | 8 | 5.49±0.11 | ∞ | t |
|  | 9 | 5.44±0.21 | ∞ | t |
|  | 10 | 4.90±0.10 | ∞ | t |
|  | 11 | 4.79±0.33 | ∞ | t |
|  | 12 | 4.68±0.21 | ∞ | t |
|  | 13 | 4.63±0.27 | ∞ | t |
|  | 14 | 4.41±0.21 | ∞ | t |
|  | 15 | 4.04±0.33 | ∞ | t |
|  | 16 | 3.66±0.18 | ∞ | t |

* m: metacentric; sm: submetacentric; st: subtelocentric; t: telocentric.
